# Editorial: New Technologies in Cancer Diagnostics and Therapeutics

**DOI:** 10.3389/fphar.2021.760833

**Published:** 2021-09-06

**Authors:** Haichang Li, Serena Li Zhao, Pascale Cohen, Dong-Hua Yang

**Affiliations:** ^1^ Department of Surgery, The Ohio State University College of Medicine, Columbus, OH, United States; ^2^ Department of Medicinal Chemistry and Pharmacognosy, The Ohio State University College of Pharmacy, Columbus, OH, United States; ^3^ University of Lyon Claude Bernard, INSERM, UMR1033 LYOS, Lyon, France; ^4^ Department of Pharmaceutical Sciences, College of Pharmacy and Health Sciences, St. John’s University, Queens, New York

**Keywords:** cancer, technologies, diagnostics, therapeutics, cancer biology

Cancer is a leading public health problem worldwide and is the second leading cause of mortality in the United States (Ferlay et al., 2021; Sung et al., 2021). The burden of cancer incidence and mortality is rapidly growing due to both the aging and the growth of the population. Despite available treatment including surgical, chemotherapeutic, radio- and immunotherapy, there is still a great need for novel diagnostic and therapeutic approaches to meet the challenges of cancer eradication.

Therefore, this research topic “*New Technologies in Cancer Diagnostics and Therapeutics*” focuses on the newly emerging areas of cancer diagnostics, drug development, and molecular signaling pathways involved in tumorigenesis and tumor development. We were excited to receive 65 contributions and finally 42 articles contributed by more than 320 authors from various countries in the fields of cancer biology, pharmacology and therapeutics were selected to be included in this topic collection.

In this topic collection, the systematic review and meta-analysis by Wen et al. summarized the use of post-diagnostic beta blocker for ovarian cancer (OC) prognosis. They screened 11 cohort studies with 20,274 OC patients. Random-effects models were used to calculate overall hazard ratios (HRs) and 95% confidence intervals (CIs). They concluded that HRs did not reveal any statistically significant associations between post-diagnostic beta-blocker use and OC prognostic characteristics, although more prospective cohort studies are necessary to further verify their results. Another three reviews included in this collection review the structures and mechanisms of proteolysis targeting chimeric (PROTACs) and describe several classes of effective PROTAC degraders (Qi et al.), advances approaches to breast cancer diagnosis and therapy (Zubair et al.), and *advances in optical aptasensors for early detection and diagnosis of various cancer types* (Zahra et al.**).**

Two case reports, one by Zhang et al., on the primary pulmonary NUT-midline carcinoma brought novel insights on the diagnosis of this type of rare tumors; the other by Wang et al. introduced using *a PD-1 inhibitor to induce the complete response of advanced bladder urothelial carcinoma*.

The discovery of long non-coding RNA (lncRNAs) is propelling the future advancement of biomarker development, and cancer diagnosis and treatment (Zhang et al., 2013; Chi et al., 2019; Jiang et al., 2019; Carlevaro-Fita et al., 2020). Zhang et al. analyzed the expression and clinical significance of lncRNA BM466146 in breast cancer and explores the role of BM466146 in immune regulation. Their findings indicated that the lncRNA BM466146 has the tumor suppressor function. Overexpression of BM466146 is associated with a better prognosis. BM466146 could regulate CXCL-13 by adsorbing hsa-miR-224-3p and inducing CD8^+^ T cells to accumulate in the tumor area which regulate immune response. Therefore, BM466146 could be a prognostic biomarker and a molecular immune target of breast cancer. Similarly, Ma et al. found that lncRNA AL161431.1 was highly expressed in pancreatic cancer cells and tissues. Knockdown of lncRNA AL161431.1 led to increased cancer cell death and cell cycle arrest. Xenograft growth of SW1990 cells with stable knockdown of lncRNA AL161431.1 in mice was significantly slower than that of SW1990 cells with scrambled control shRNA. Yu et al. investigated the role of LncRNA SNHG8 in regulating diffuse large B-cell lymphoma (DLBCL) cells and uncover its underlying mechanism. Their findings suggested that lncRNA SNHG8 exerted a cancer-promoting effect on DLBCL via targeting miR-335-5p. Liao et al. characterize the molecular mechanism of LINC00667 in nasopharyngeal carcinoma (NPC) progression and found that LINC00667 could be a diagnostic and therapeutic target for NPC patients. Luo et al. investigated the potential prognostic role of autophagy-related lncRNA in patients with bladder cancer (BC) and identified 15 autophagy-related lncRNAs have the prognostic potential for BC, and may play key roles in BC biology. Zheng et al. identified a survival-related gene-based ceRNA network using the WGCNA algorithm, and the constructed lncRNA–miRNA–mRNA ceRNA interactive network to provide novel insights into the treatment of gastric cancer. Taken together, these lncRNAs could be able to predict clinical outcomes, holding great promise in future clinical applications.

Novel targets and biomarkers are essential components in drug developments and treatments, particularly in this era of targeted therapies. Tremendous efforts are being made to interpret the mechanisms of cancer development with the aim of discovery of novel drugs. Li et al. identified MOF as an oncogene in thyroid cancer and found that the expression of MOF was significantly upregulated in most thyroid cancer tissue samples and cell lines. MOF is a well-known histone acetyltransferase, which is involved in diverse biological processes, such as gene transcription, cell cycle, early embryonic development and tumorigenesis. Their findings demonstrated that MOF played an oncogenic role in the development and progression of thyroid cancer and may be a potential novel target for the treatment of thyroid cancer. Bellanger et al. provided insights into the biological and clinical significance of the novel exon4-skipping isoform of the well-established oncogene ZNF217 in breast cancer. They revealed that, in the Luminal subclass, a dual signature combining the expression levels of these two isoforms may serve as a novel prognostic biomarker allowing better stratification of breast cancers with good prognosis and aiding clinicians in therapeutic decisions. They also identified that ZNF217-ΔE4 protein drives cell aggressiveness and that a close interplay exists between the ZNF217-WT and ZNF217-ΔE4 isoforms. Wang et al. identified prognostic biomarkers for patients with EGFR-WT non-small-cell lung cancer and confirmed a novel potential role for HDAC is in the clinical management of EGFR-WT patients. Liu et al. investigated transient receptor potential melastatin-related 7 (TRPM7) mediated glioma stemness. They found that TRPM7 mRNA expression is significantly increased in anaplastic astrocytoma, diffuse astrocytoma, and GBM patients compared to that in healthy brain tissue controls. TRPM7 expression in GBM cells was found to be positively correlated with Notch1 signaling activity and CD133 and ALDH1 expression. Moreover, they found that targeting Notch1 compromises the TRPM7-induced growth and proliferation of glioma cells. Yang et al. demonstrated that *targeting the B7-H3 Immune checkpoint with specific CAR-engineered NK-92 cells exhibits potent cytotoxicity against non-small cell lung cancer*. Wang et al. investigated the role of a novel Indole derivative, named LCT-3d, in inhibiting the growth of gastric cancer cells. LCT-3d modulated the mitochondrial-related proteins and Cleaved-Caspases 3/9, to induce cell apoptosis. Their findings suggested that LCT-3d induces apoptosis via DR5-mediated mitochondrial apoptotic pathway in gastric cancer cells. LCT-3d could be a novel lead compound for development of anti-cancer activity in gastric cancer.

Since the development of drug resistance is a major contributor towards the failure of chemotherapeutic regimens, efforts have been made to develop novel inhibitors that can combat drug resistance and sensitize cancer cells to chemotherapy (Vasan et al., 2019). Yang et al. investigated the interaction of OTS964, a potent T-LAK cell-originated protein kinase (TOPK) inhibitor and multidrug resistance (MDR)-associated ATP-binding cassette sub-family G member 2 (ABCG2). They found that the OTS964 is susceptible to ABCG2-mediated drug resistance, and this effect can be antagonized by a known ABCG2 inhibitor. These findings may serve as a valuable foundation for follow-up clinical investigation on potential use of OTS964. Tumoral microenvironmental is a critical influencing factor for multiple anti-cancer treatments. In this topic collection, Li et al. demonstrated that acidic pH attenuates the cytotoxic activity of pharmacologic ascorbic acid by inhibiting ascorbic acid (AA) uptake in PCa cells. Additionally, they found that the cancer cell-selective toxicity of AA depends on ROS. *In vivo*, the combination of AA and bicarbonate could provide a significantly better therapeutic outcome in comparison with controls or AA single treated mice. Chimeric antigen receptor (CAR)-modified natural killer (NK) cell therapy represents a kind of promising anti-cancer treatment. Yang et al. constructed the second-generation CAR consisting of 4-1BB costimulatory signal domain and a CD3ζ domain, which is similar to the clinically used anti-CD19 CAR-T cells. Compared to unmodified NK-92MI cells, the activity and cytotoxicity of CAR-modified NK-92MI cells were significantly enhanced both *in vitro* and *in vivo*. Those results demonstrate that redirection of anti-B7-H3 CAR promotes activation and antitumor cytotoxicity of NK cells.

In conclusion, this research topic highlights multiple aspects of the emergence of novel modalities that could be of potential application on cancer diagnosis and treatment.
